# Ferroptosis: a new hunter of hepatocellular carcinoma

**DOI:** 10.1038/s41420-024-01863-1

**Published:** 2024-03-13

**Authors:** Yulang Jiang, Yongxin Yu, Ziyang Pan, Christian Glandorff, Mingyu Sun

**Affiliations:** 1 Shuguang Hospital Affiliated to Shanghai University of Traditional Chinese Medicine, Shanghai, 201203 China; 2 Shanghai University of Traditional Chinese Medicine, Shanghai, 201203 China; 3 Key Laboratory of Liver and Kidney Diseases, Institute of Liver Diseases, Shuguang Hospital Affiliated to Shanghai University of Traditional Chinese Medicine, Shanghai, 201203 China; 4 University Clinic of Hamburg at the HanseMerkur Center of TCM, Hamburg, Germany

**Keywords:** Tumour biomarkers, Targeted therapies

## Abstract

Ferroptosis is an iron ion-dependent, regulatory cell death modality driven by intracellular lipid peroxidation that plays a key role in the development of HCC. Studies have shown that various clinical agents (e.g., sorafenib) have ferroptosis inducer-like effects and can exert therapeutic effects by modulating different key factors in the ferroptosis pathway. This implies that targeting tumor cell ferroptosis may be a very promising strategy for tumor therapy. In this paper, we summarize the prerequisites and defense systems for the occurrence of ferroptosis and the regulatory targets of drug-mediated ferroptosis action in HCC, the differences and connections between ferroptosis and other programmed cell deaths. We aim to summarize the theoretical basis, classical inducers of ferroptosis and research progress of ferroptosis in HCC cells, clued to the treatment of HCC by regulating ferroptosis network. Further investigation of the specific mechanisms of ferroptosis and the development of hepatocellular carcinoma and interventions at different stages of hepatocellular carcinoma will help us to deepen our understanding of hepatocellular carcinoma, with a view to providing new and more precise preventive as well as therapeutic measures for patients.

## Facts

- Ferroptosis plays an extremely important role in the development of hepatocellular carcinoma, and precise targeting of tumor cells to induce ferroptosis is a new way forward in the treatment of cancer.
- Ferroptosis occurs as a metabolic mode of cell death involving lipid metabolism, amino acid metabolism, iron metabolism, glutathione metabolism, etc., and an imbalance in any of these metabolisms affects the extent of cellular ferroptosis.
- Natural defense mechanisms against ferroptosis exist within the cell, consisting mainly of enzymatic and non-enzymatic antioxidant mechanisms.
- Studies have shown that many ferroptosis-regulated genes are widely involved in the development of hepatocellular carcinoma, and the resistance problem of sorafenib, the first-line drug in hepatocellular carcinoma clinics, is also inextricably linked to ferroptosis.

## Questions

- What is the ultimate executioner of ferroptosis?
- What is the normal physiological function of ferroptosis?
- How to accurately target ferroptosis in tumor cells and reduce the level of ferroptosis in normal cells, and whether there are recognition molecules on the surface of different cells with different degrees of sensitivity to ferroptosis?
- How ferroptosis and other modes of cell death interact and are regulated in tumor therapy, or how much ferroptosis is involved?

## Introduction

Ferroptosis was proposed in 2012 as an iron-dependent, non-apoptotic form of cellular demise instigated by lipid peroxidation [1]. The occurrence of ferroptosis is dependent on the accumulation of iron in cells and lipid peroxidation. Multiple intracellular molecules regulate the onset of ferroptosis by affecting intracellular iron levels and lipid peroxidation status. Cells undergoing ferroptosis exhibit morphological features that are more distinctive from other programmed cell death modalities such as apoptosis, autophagy, necroptosis, and pyroptosis. The main features of ferroptosis and apoptosis, necroptosis, pyroptosis, and autophagy are shown in Table 1. The most predominant morphological changes of ferroptosis include atrophy of mitochondria and reduction of cristae whereas the nucleus usually remains intact. In contrast, apoptosis usually involves fragmentation and margination of chromatin, as well as the production of apoptotic vesicles and plasma membrane vesicles [2]. Programmed cellular necrosis has been shown to occur with cell swelling and the production of plasma membrane fragments that are eventually released from the plasma membrane in the form of vesicles [3]. When cells undergo autophagy, the intracellular lysosomes form a double membrane, While the membrane structure and nuclear morphology are more normal [4]. Another form of cell death is pyroptosis, which is heavily dependent on the gasdermin D protein. Pyroptotic cells swell and expand until the cell membrane ruptures, leading to the release of cellular contents and the activation of a strong inflammatory response [5]. All of these different modes of cell death are significantly different from ferroptosis. By comparing ferroptosis with other forms of cell death, it can help us better understand the mechanism of ferroptosis occurrence and its unique features. In summary, ferroptosis as a new form of programmed cell death is characterized by iron dependence and increased lipid peroxidation. Ferroptosis plays an important role in cell proliferation, differentiation, cycling and senescence [6]. Ferroptosis can be induced to occur by structurally distinct small molecules (e.g., erastin, sulfasalazine, and RSL3) or inhibited by lipophilic antioxidants (e.g., CoQ10, vitamin E, ferrostatins, and liproxstatins). Elevated intracellular iron ion concentrations and deficiency of the antioxidant GSH lead to cellular lipid peroxidation, which results in cellular ferroptosis [7, 8].

**Table 1:** Comparison between different modes of programmed cell death.

Cell death mode	Morphological characteristics	triggering factor	Inhibitors	Regulatory mechanism	Key target
Apoptosis	Cell membranes and organelles are relatively intact, cells are wrinkled, and nuclei are consolidated [175]	Radiation, toxins, hypoxia, endoplasmic reticulum stress, DNA damage, etc [176]	Z-VAD-FMK [177]	Exogenous pathway: triggered by the death receptor Fas and the tumor necrosis receptor family TNF-R. Junction protein (FADD/TRADD) Pro-Caspase-8, leads to the formation of death-inducing signaling complex (DISC), Caspase-8 oligomerizes and is activated by autocatalysis, activated Caspase-8 induces apoptosis. Endogenous pathway: also known as the mitochondrial pathway to apoptosis, is the main site of apoptosis and participates in most of the processes that regulate apoptosis. When cells are subjected to endogenous stress, pro-apoptotic factors within the mitochondrial membrane are released into the cytoplasm, activating the cellular mitochondrial apoptotic pathway, and causing cell death. These pro-apoptotic factors include cytochrome C (CytC), apoptosis-inducing factor (AIF), cysteine aspartate protein hydrolase activator (Smac/DIABLO), and apoptotic protease activator (Apaf-1). Cytochrome C is released from the mitochondria into the cytoplasm, where it binds to Apaf-1 to form the apoptotic complex, which further activates Caspase-9 and triggers a cascade reaction of caspases, ultimately leading to the segmentation of the cell into many apoptotic vesicles [178–181]	caspase-8/9/3, Bcl-2, Bcl-xl, P53, HSP70
Necroptosis	Cell rounding, cytoplasmic swelling, organelle expansion, nucleus lysis, cell membrane disintegration [182]	Inflammatory factors, bacteria, viruses, immune response [183]	Necrostatin-1, Necrostatin-7, MLKL-IN-3 [184, 185]	It consists of two receptor-interacting protein kinases (RIPK1 and RIPK3) and a mixed-spectrum kinase structural domain-like (MLKL). RIPK3 regulates the phosphorylation of MLKL, induces its oligomerization, translocates to the plasma membrane, and generates a pore complex at the plasma membrane, resulting in the secretion of DAMPs (damage-associated molecular patterns) [186]	TNF, TNFR1-TRADD, RIP1, RIP3-MLKL
Pyroptosis	Cells swelling, expanding, rupture of cell membranes [187]	Inflammatory factors, bacteria, viruses, immune response [188]	Antcin A, VX765 [189]	Classical pathway: cells assemble into inflammatory vesicles in response to inflammatory inducers, inflammatory vesicles convene and activate caspase-1, and activated caspase-1 cleaves GSDMD mediating the creation of membrane pores and subsequent release of contents. Non-classical pathway: caspase-4 and 5 are activated to cleave GSDMD in response to LPS stimulation [190]	Caspase-1, Gasdermin D, IL-1β, NLRP3
Autophagy	Cytoplasm is vacuolated, chromatin does not aggregate, and membrane structure is intact [4]	External stress, starvation, hypoxia, endoplasmic reticulum stress [191]	3-MA, Chloroquine [192, 193]	Autophagy is an intracellular process of self-degradation and recycling that involves several conserved ATG (autophagy-related) proteins. The initiation and phagocytic vesicle formation phases of autophagy involve the VPS34 complex and the ULK1 complex, which are positive regulators. The extension of phagocytic vesicles and the formation of autophagosomes require the involvement of two ubiquitin-like coupling pathways, including ATG5-ATG12 coupling and LC3 processing. Extension and completion of the autophagosome membrane is not possible without LC3B-II. After fusion of the autophagosome with the lysosome, small molecules produced by degradation, particularly amino acids, are translocated back to the cytoplasm for protein synthesis and to maintain cellular function under starvation conditions [194, 195]	ATG, LC3, P62
Ferroptosis	Mitochondria are small and crumpled, cristae are reduced, nuclei are normal, and cell membranes are ruptured [196]	Iron ion accumulation, reactive oxygen species accumulation, lipid metabolism imbalance [197]	Ferrostatin-1, Liprostatin-1, DFO, NAC, Trolox [198–201]	In the presence of divalent iron or ester oxygenase, which catalyzes the high expression of unsaturated fatty acids on the cell membrane, lipid peroxidation occurs, resulting in the induction of cell death; in addition, a decrease in GPX4, a core enzyme in the regulation of the antioxidant system (the glutathione system), is also manifested [195–202]	SLC7A11, GPX4, FSP1, ACSL4, PTGS2, NRF2

Morphological alterations, triggering factors, classical inhibitors, molecular mechanisms and core targets of different programmed cell deaths.

The principal regulatory mechanisms orchestrating ferroptosis encompass lipid peroxidation, amino acid metabolism, oxidative stress, iron ion homeostasis, and diverse organelles, all constituting prerequisites for the manifestation of ferroptosis to occur. Naturally, the body is equipped with a ferroptosis defense system to safeguard cells, primarily including (1) Cystine/System Xc^−^/GSH/GPX4, (2) FSP1-CoQ10-NAD(P)H, (3) DHODH-CoQ10-CoQH2, (4) GCH1/BH4/DHFR, and (5) FSP1/ ESCRT-III [9]. The body maintains a dynamic equilibrium of cellular state by modulating both the promotive and resistive factors of ferroptosis. These essential prerequisites and defense mechanisms for the occurrence of ferroptosis inherently convey that ferroptosis constitutes a metabolic form of cell demise. While the physiological relevance of ferroptosis remains a subject of debate, its inseparable connection with hepatocellular carcinoma is undeniable.

Primary hepatic carcinoma stands as the foremost neoplastic manifestation within the liver, ranking as the fourth principal cause of global cancer-related mortality [10, 11]. According to the Barcelona Clinic Liver Cancer classification, the majority of patients present at an intermediate to advanced disease stage upon diagnosis, foreclosing the prospect of radical surgical intervention [12]. The current first-line drugs for HCC patients are the multi-kinase inhibitors sorafenib and lenvatinib. However, a multicenter randomized controlled clinical trial showed that sorafenib only prolonged the median survival of patients with HCC by 10 months and lenvatinib by 15 months [13]. Unfortunately, despite this, patients still lose the effectiveness of the drug after a few months of treatment due to drug resistance [14–16].

Certain investigations have identified a propensity for iron ion accumulation and lipid peroxide overload in HCC. For certain tissue sources, specific cell differentiation states, and certain types of HCC cells, although they are resistant to other types of induced cell death, they are extremely sensitive to ferroptosis inducers. Sorafenib is the most widely studied of these drugs. Some studies have found that sorafenib induces ferroptosis in some tumor cells and that ferroptosis regulatory proteins also affect the sensitivity of HCC to the sorafenib [17]. Secondly, in the tumor microenvironment, immune cells and their factors affect the susceptibility of tumor cells to ferroptosis [18], while ferroptosis of tumor cells triggers the host to mount an anti-tumor immune response [19]. Therefore, targeting ferroptosis in tumor cells would be a promising therapeutic strategy for tumor treatment and combined with immunotherapy to produce coordinated antitumor effects. The following will describe the discovery of ferroptosis targets in HCC in recent years [20].

The regulatory frameworks governing networks of ferroptosis in liver cancer have been discovered to possess a high degree of richness, bordering on extraordinariness. including P53, P62-Keap1-Nrf2-ARE, Rb, MT-1G, S1R, CER, ACSL4, CISD1, HNF4A/HIC1, etc. The mechanisms by which these targets function in the ferroptosis network and how they regulate the drug sensitivity to ferroptosis in HCC cells will be expounded upon in meticulous detail [21].

## Prerequisites for ferroptosis

### Lipid peroxidation

The cellular membrane is a barrier that prevents extracellular substances from freely entering the cell, it ensures the stability of the intracellular environment, and lipids, as the main components of the cellular membrane, distinguish the biochemical processes inside the cell from the external environment [22]. The composition and content of lipids in the membrane vary depending on the cell type, organelles, and membrane leaflets [23]. Disruption of membrane integrity is essential for the execution of all forms of regulated cell death [24].

The operative molecules of cellular ferroptosis are specific lipid peroxides and peroxyl radicals containing polyunsaturated lipid chains. These lipids can form lipid peroxides by autoxidation or enzymatic catalysis, which are subsequently converted to lipid peroxyl radicals in the presence of divalent iron ions, thereby affecting the fluidity, integrity and stability of the cell membrane [25]. Disruption of cell membrane integrity is the underlying driver of ferroptosis occurrence.

Lipid metabolomics reveals that polyunsaturated fatty acids such as arachidonic acid (AA) or Adrenic Acid (AdA) are the most oxidized lipids during ferroptosis and are regulated by three synthetases [26]. Acyl-CoA synthetase long-chain familymember 4 (ACSL4) catalyzes the conversion of AA or AdA to AA-CoA and AdA-CoA [27]. Subsequently, Lys phosphatidylcholine acyltransferase 3 (LPCAT3) esterifies it to phosphatidylethanolamines (PEs) to form AA-PE and AdA-PE [28], and finally oxidized to PE-AA-OOH and PE-AdA-OOH by polyunsaturated fatty acid lipoxygenase 15 (ALOX15) [29]. And Glutathione peroxidase 4 (GPX4) can convert these lipid peroxides back to the corresponding alcohols, thereby reducing the level of intracytoplasmic lipid peroxides. [30]. Studies also showed that knockdown or inhibition of all three of these synthases inhibited the development of ferroptosis [31]. The excessive accumulation of specific lipid peroxides directly precipitates ferroptosis, provoking disturbances such as cell membrane disruption, cellular swelling and mitochondrial fragmentation, ultimately culminating in the manifestation of ferroptosis.

Since polyunsaturated fatty acids (PUFA) promote ferroptosis and monounsaturated fatty acids (MUFA) inhibit ferroptosis [32], inducing the conversion of monounsaturated fatty acids to polyunsaturated fatty acids to enhance the susceptibility to ferroptosis in hepatocellular carcinoma may be a proven strategy.

### Amino acid metabolism

Amino acid metabolism sustains human life by providing essential elements such as proteins, energy substrates, glutathione, and neurotransmitters for human life. It intricately governs ferroptosis by regulating several lipid antioxidant systems. Even before the discovery of ferroptosis, scientists observed that cystine deficiency in the culture medium led to cell death, and that cells could only grow at extremely high densities. Further research found that, cysteine amino acid metabolism is most closely related to ferroptosis, which is inhibited through metabolic pathways such as synthesis of glutathione [33], glutathione peroxidase 4 [34], iron-sulfur cluster [35], and hydrogen sulfide [36]. Methionine can regulate ferroptosis through the sulfur transfer pathway and polyamine synthesis. The mevalonate pathway is involved in the synthesis of selenocysteine and coenzyme Q10 (CoQ10), products that are catalytic centres for GPX4 and reducing agents for the inhibition of lipid peroxidation, respectively [37–40]. In contrast, glutamine catabolism also promotes ferroptosis induced by cysteine deprivation [41].

In conclusion, amino acid anabolism intricately participates in the complex network governing ferroptosis regulation, where GSH levels may be a hub for the regulation of ferroptosis sensitivity.

### Oxidative stress and ferroptosis

Oxidative stress is cellular and tissue damage caused by the production of reactive oxygen species (ROS) in the organism that exceed their scavenging effect [42]. Essentially, ferroptosis is caused by an imbalance in iron-mediated production and scavenging of ROS resulting in cell death due to the accumulation of lethal lipid peroxides [43]. This disequilibrium in the dynamic process is the direct cause of ferroptosis [21]. ROS play a pivotal role in cellular signal transduction and tissue homeostasis [44]. The primary ROS components encompass the anion(O_2_^−^), peroxides (H_2_O_2_) and LOOH, and free radicals (HO· and LO·). Mitochondria serve as the main source of ROS [45]. Mitochondria produce a large amount of free ROS in the normal metabolism of electron transport chain and cell energy supply, and endogenous ROS can also be released by microsomes, nicotinamide adenine dinucleotide phosphate (NADPH) oxidase, xanthine oxidase, peroxidase, etc. There is a homeostatic ROS scavenging system in the body, which can remove excessive ROS in the body. The body’s clearance of ROS is mainly dependent on the body’s antioxidant system, including Superoxide dismutase (SOD) [46], Catalase (CAT), GPX4, etc. [47].

The significance of oxidative stress in ferroptosis is further demonstrated by the fact that oxidative stress is involved in the whole process of ferroptosis, and the antioxidant system mitigates ferroptosis by scavenging ROS [48, 49].

### Iron ion homeostasis

Normal intracellular iron levels play an irreplaceable role in maintaining normal cellular physiological functions. Abnormalities in any of the various aspects of iron metabolism, involving, for example, iron absorption, storage, conversion, utilization, distribution and excretion, can lead to an imbalance in the intracellular iron ion content, thus affecting the overall cellular state and the function of the organism [50–52]. As the main form of intracellular iron presence, the relative conversion of ferrous and ferric ions, as the primary forms of intracellular iron, directly or indirectly influences cellular metabolism and viability [8, 51]. The liver, as the largest iron storage organ in the body, plays an irreplaceable role in the regulation of iron metabolism [53, 54]. Meanwhile, the change of iron content in the liver is also closely related to the physiological state of the liver [50, 55].

The main forms of iron present in the body include trivalent iron and divalent iron [56]. Trivalent iron can be reduced to divalent iron by duodenal cytochrome b and can be transported to the duodenal epithelium by divalent metal transporter 1 (DMT1) [57]. transferrin-bound (TF) iron uptake mediated by Transferrin Receptor 1 (TFR1), or non-TF-bound iron uptake mediated by Solute carrier family 39 (SLC39A14) is the major intracellular iron acquisition pathway [58, 59]. Trivalent iron ions can also be converted to divalent ferrous ions by Six-Transmembrane Epithelial Antigen of Prostate 3 (STEAP3) [60], which excretes iron required for normal physiological functions, and the rest is partially transported out of the membrane by ferroportin (FPN) [61, 62], consisting of ferritin heavy chain 1 (FTH1) and ferritin light chain (FTL), and stored in tissues and organs, such as the liver [63]. Additionally, heme degradation and NCOA4-mediated ferritin autophagy can degrade ferritin, releasing iron into the labile iron pool (LIP) [64, 65], subsequently sensitizing cells to iron-induced atrophy via the Fenton response. The intracellular iron content is also influenced by hepcidin, which at low levels rapidly and stably transports iron into the plasma, while at high levels, FPN is inactivated and iron entry into the plasma is restricted [66, 67]. The amount of ferrous ions in the unstable iron pool is a key factor in determining the sensitivity of ferrous ions to iron atrophy. It has also been found that such as SLC39A8 can also exercise the function of iron transport [68].

Cellular ferroptosis, triggered by intracellular iron accumulation, emerges as a pathogenic factor in HCC. Consequently, considering iron chelators to impede ferroptosis may present a promising therapeutic approach for associated diseases [8, 69–73].

### Cell organelles and ferroptosis

Various organelles assume significant roles in the development of ferroptosis. Mitochondria, as the epicenter of energy metabolism, intricately regulate amino acid, carbohydrate, and lipid metabolism, influencing ferroptosis. Simultaneously, they modulate ferroptosis through ROS and may impact iron metabolism [74–76]. The release of iron ions by lysosomes through the degradation of ferritin mediated by autophagy increases intracellular free iron ion content, thereby promoting ferroptosis [6, 77, 78]. The rich lipid content of the endoplasmic reticulum is the main substrate source of ferroptosis, and the synthesis of fatty acids is mainly completed in the endoplasmic reticulum. Secondly, ferroptosis inducers can activate endoplasmic reticulum stress [79–82]. Peroxisomes regulate ferroptosis by regulating the antioxidant system. Further studies on lipidomics have found that peroxisomes can also regulate ferroptosis by regulating the synthesis of PUFA-ePL, and the sensitivity of cells to ferroptosis is also closely related to PUFA-ePL [83–85].

**Fig. 1:**
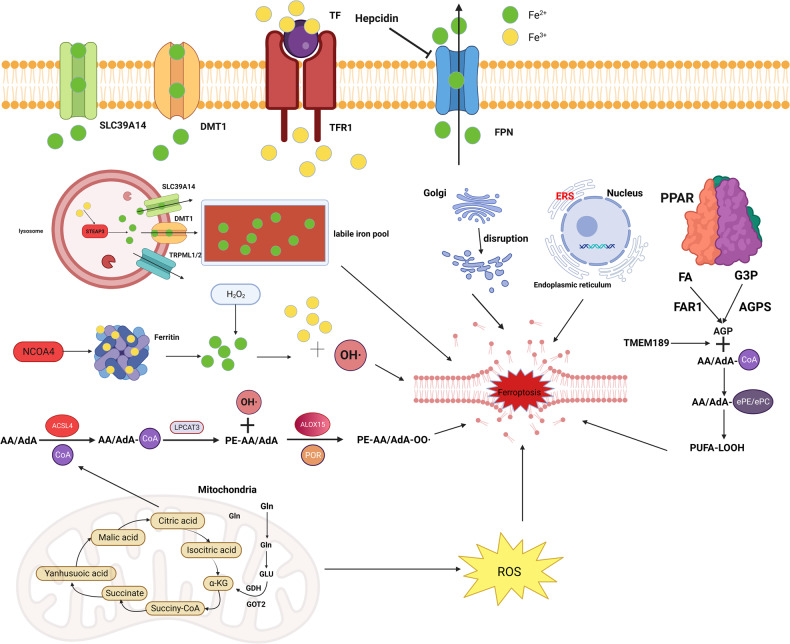
Drivers of ferroptosis. The initiators of ferroptosis include lipid metabolism and iron metabolism. In lipid metabolism, adrenoic acid and arachidonic acid in a series of enzymatic reactions culminate in the production of PUFA to promote ferroptosis. In iron metabolism, the binding of transferrin (TF) and transferrin receptor (TFRC) on the membrane drives extracellular Fe^3+^ into the cytosol, followed by the reduction of Fe^3+^ to Fe^2+^ by STEAP3, which is transported between different organelles under the action of SLC39A14, DMT1, and TRPML1/2, and finally sinks into the Lip. Also, various organelles are extensively involved in ferroptosis; the tricarboxylic acid cycle in mitochondria generates enormous amounts of ROS, and metabolic activity or oxidative stress in the Golgi, endoplasmic reticulum, and lysosomes can trigger ferroptosis.

In summary, the regulation of ferroptosis requires the coordinated operation of multiple organelles, so understanding how these organelles interact has implications for the development of clinical drugs for ferroptosis (Fig. 1).

## Ferroptosis’s defense system

### Cystine/System Xc-/GSH/GPX4

The Xc^−^ system, a sodium-independent reverse transporter for cystine and glutamate, comprises integral subunits: the light chain, Solute Carrier Family 7 Member 11 (SLC7A11) (also known as xCT) [86], serving as the main functional subunit, and the heavy chain subunit Solute Carrier Family 3, Member 2 (SLC3A2), also known as (4F2hc), acting as the molecular chaperone [87, 88]. This system facilitates the transfer of cystine from the extracellular to intracellular environment in a 1:1 ratio, with rapid conversion of cystine to cysteine upon entry. In the initial step, γ-GCS catalyzes the dehydration condensation of cysteine with glutamate to γ-glutamyl cysteine. In the second step, γ-glutamyl cysteine undergoes dehydration condensation with glycine again to glutathione catalyzed by GS. Where γ-GCS is the rate-limiting enzyme for the reaction and cysteine is the rate-limiting substrate for the reaction [89, 90].

GPX4 is an crucial negative regulator of lipid peroxidation in living organisms [91]. GPX4 protects cells from ferroptosis induced by lipid peroxidation by catalyzing the conversion of harmful intracellular lipid peroxides to harmless lipid alcohol compounds. GPX4 uses GSH as a cofactor to reduce peroxides (e.g., R-OOH) to the corresponding alcohols (R-OH) and also to reduce free of hydrogen peroxide to water, reducing the accumulation of toxic free radicals (e.g., RO-) [49, 92].

Functioning as a cell protector, GPX4 employs reduced GSH as a substrate to act as a cell protector and resist oxidative stress. During the reduction of peroxides (L-OOH) by GPX4, the oxidized GSSG produced can be reduced by glutathione reductase and reduced coenzyme ii (NADPH/H+) to regenerate reduced GSH, leading to the recycling of GSH [93]. Although GPX4 can catalyze the reduction of hydrogen peroxide, organic peroxides and lipid peroxides [94]. However, GPX4 prefers lipid peroxides. Thus, GPX4 is the main defender of cellular ferroptosis in living organisms [95, 96].

### FSP1-CoQ10-NAD (P)H

Ferroptosis suppressor protein 1 (FSP1), also identified as Mitochondrial apoptosis-inducing factor 2 (AIFM2) [97], is a flavoprotein oxidoreductase encoded by the AIFM2 gene. Initially discovered in 2002, FSP1 was first considered a P53-responsive gene.

Initially it was thought that ferroptosis was regulated only by GPX4, which reduces hydroperoxides, and by antioxidants, which trap free radicals. However, one study found that certain tumor cells were still able to survive and proliferate after GPX4 deletion. This suggests that there may be a parallel ferroptosis inhibition axis independent of the GPX4 axis.

Two papers published back-to-back in Nature 2019 suggest that FSP1 overexpression in cells significantly protects them from ferroptosis-inducing factors and that this process is not dependent on GPX4 [98, 99]. At the plasma membrane, FSP1 catalyzes the reduction of coenzyme Q10 (also known as ubiquinone) to panthenol-10, a process that requires the involvement of NAD(P)H, a lipid-soluble radical-trapping antioxidant that prevents ferroptosis caused by lipid peroxidation damage, and this biological function is independent of the level of intracellular glutathione [99, 100].

### DHODH- CoQ10-CoQH2

Mitochondria are organelles wrapped by dual membranes, the inner and outer membrane, function as the main sites of aerobic respiration, where the inner membrane orchestrates electron transfer, generating large amounts of ROS. Dihydroorotate dehydrogenase (DHODH), located in the inner mitochondrial membrane, is responsible for catalyzing the fourth step of the pyrimidine nucleotide synthesis pathway, namely the oxidation of dihydroorotate acid to orotate acid (OA), while CoQ10 in the inner membrane receives electrons to be reduced to CoQH2 [101].

In addition to the synthesis of pyrimidine nucleotides, DHODH was found to inhibit ferroptosis in mitochondria by producing CoQH2 in the inner mitochondrial membrane because CoQH2 can act as a radical trapping antioxidant to prevent lipid peroxidation and thus inhibit ferroptosis [102].

Further studies revealed that mechanistic studies confirmed that DHODH could only perform its ferroptosis inhibitory function when localized in mitochondria and that this function was dependent on its enzymatic activity. Subsequently, it was found that co-inactivation of DHODH and mitochondrial GPX4 led to mitochondrial lipid peroxidation and that BQR treatment significantly increased the intracellular CoQ10/CoQH2 ratio [103], suggesting that DHODH inhibits mitochondrial lipid peroxidation by reducing CoQ10 to CoQH2 in concert with mitochondrial GPX4. Notably, the cell membrane, composed of cytosolic GPX4 and FSP1, constitutes a distinct ferroptosis defense system [104].

### GCH1/BH4/BH2

A GPX4-independent ferroptosis inhibition was identified through a CRISPR genome-wide screen: the GTP Cyclohydrolase 1 (GCH1), GCH1 is another important regulator of ferroptosis. It catalyzes the conversion of dihydrobiopterin (BH2) to tetrahydrobiopterin (BH4). BH4 is a cofactor for aromatic amino acid hydroxylase and other enzymes, and GCH1 mediation is the rate-limiting reaction in the BH4 biosynthesis pathway. BH4 is another radical-trapping antioxidant that can capture free radicals from lipid peroxidation. Its recycling requires the involvement of dihydrofolate reductase (DHFR), and its inhibition of ferroptosis seems to be independent of the action of its cofactor. Ferroptosis was inhibited by the generation of BH4 as a free radical trapping antioxidant, and by GCH1-mediated production of CoQH2 and PLs containing two PUFA tails. However, the specific subcellular compartment in which the GCH1-BH4 system works remains to be determined. BH4 mechanism [105], a free radical-trapping antioxidant whose recycling requires the involvement of the DHFR, and thus if blocked, the DHFR could potentially collaborate with GPX4 inhibitors to induce ferroptosis [106].

### FSP1/ESCRT-III

**Fig. 2:**
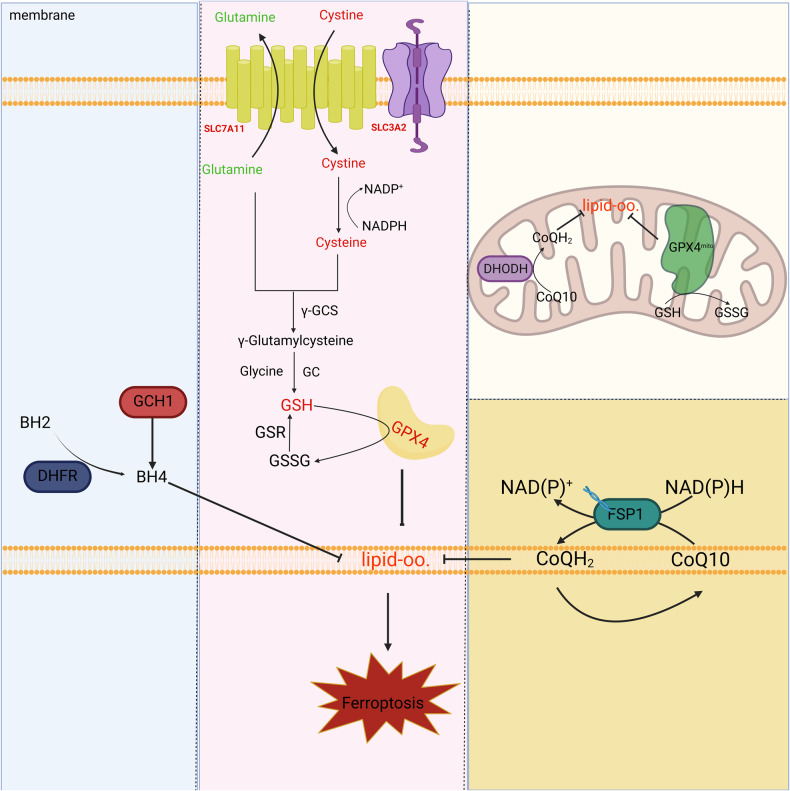
Defenders of ferroptosis. (1) Cystine is transported into the cytosol via the cystine/glutamate reverse transporter and metabolized to cysteine. Cysteine and intracellular glutamate are metabolized by glutamate. Cysteine ligase (GCL) catalyses the formation of Y-glutamate cysteine. The latter is catalyzed by glutathione synthetase (GS) and glycine (Gly) to produce glutathione. Glutathione peroxidase 4, assisted by the cofactor glutathione, inhibits the accumulation of phospholipid hydroperoxides (PL00H) and ultimately inhibits ferroptosis. (2) Coenzyme Q10 generates its reduced form CoQ10-H2 in the presence of FSP1. (3) GCH1 promotes BH4 biosynthesis reduces phospholipid hydroperoxide accumulation inhibits ferroptosis. (4) DHODH, localized in the mitochondria, oxidizes dihydroorotate (DHO) to orotate (OA) while transferring electrons to ubiquinone in the inner mitochondrial membrane for reduction to ubiquinol.

A 2020 study reported that FSP1-deficient tumor cells displayed heightened sensitivity to cell death induced by ferroptosis inducers, but the addition of exogenous CoQ10 did not reverse the cell death induced by FSP1 deficiency [107, 108]. In contrast, investigators have identified a novel mechanism of action of FSP1-mediated inhibition of ferroptosis by the ESCRT that is independent of CoQ10 and its downstream inhibition of lipid peroxidation [109]. Both in vivo and in vitro experiments demonstrated that inhibition of gene expression of the constituent proteins of the ESCRT-III complex significantly enhanced the level of ferroptosis in tumor cells [110]. However, the interaction of FSP1/ESCRT-III and the specific mechanism of action of this pathway still need more studies to support and elucidate (Fig. 2).

## Regulators of ferroptosis in HCC

### P53

TP53 stands as the most extensively studied and frequently mutated tumor suppressor gene in human tumors [111]. P53 acts as a double-edged sword in ferroptosis. P53 acts as a transcription factor regulating the levels of multiple ferroptosis-related genes to promote or inhibit ferroptosis.

Initially, P53 is involved in the regulation of the ferroptosis process as a transcriptional repressor of SLC7A11, inhibiting cysteine uptake and consequently GSH production and ROS-mediated increase in cellular ferroptosis, a molecular cascade reaction that may contribute to the anti-cancer effects of P53 [112]. This specific induction is mediated by a P53 mutant with an acetylation-deficient phenotype, P53KR [113]. This mutant has a strong inhibitory effect on SLC7A11 but has little inhibitory effect on other known target genes of P53 [114]. Recent studies have also shown that SLC7A11 is highly expressed in tumor cells and thus promotes tumor growth and proliferation in part by suppressing the onset of ferroptosis. Non-synonymous single nucleotide mutations in P53 codon 47, S47, do not affect most P53 functions, but their tumor suppressive effects are impaired, and S47 heterozygous and pure mice are significantly more susceptible to the liver and other cancers, which may be related to impaired negative regulation of SLC7A11 by S47, consequently inhibiting ferroptosis [115–119].

Glutaminase 2 (GLS2) is the key enzyme in the liver that catalyzes the conversion of glutamine to glutamate. One study found that the use of ferroptosis inhibitors and glutaminolysis inhibitors inhibited erastin-induced ferroptosis, resulting in an increase in intracellular GSH and a decrease in intracellular ROS content, thus finding that glutamine and GLS2 are required for the development of cellular ferroptosis. It has also been found that upregulation of GLS2 expression contributes to ferroptosis caused by aerobic glycolysis rather than oxidative phosphorylation [120].

Prostaglandin Endoperoxide Synthase 2 (PTGS2), a key enzyme in the initiation step of prostaglandin (PG) synthesis in organisms, can regulate cellular sensitivity to ferroptosis by regulating the levels of key intracellular membrane phospholipids PE.

When ferroptosis inhibitors such as GLS2 and erastin were applied to P53 wild-type cells, the cells exhibited upregulated PTGS2 gene expression and underwent ferroptosis, but when P53-deficient cells were induced with GLS2 and erastin, the PTGS2 gene expression level was unchanged and ferroptosis did not occur. Therefore, P53 is required for the upregulation of PTGS2 expression and is directly associated with ferroptosis, and PTGS2 has been widely used as a marker of ferroptosis occurrence [121, 122].

Spermidine/spermine N1-acetyltransferase 1 (SAT1) can catalyze the acetylation reaction of spermidine and spermine using CoA. Abnormal polyamine metabolism is closely related to tumors. SAT1 activation can induce ferroptosis, while P53 can induce SAT1 transcriptional expression to promote lipid peroxidation and ROS-induced ferroptosis, and silencing SAT1 diminishes ROS-induced cellular ferroptosis in wild-type P53 cells [123, 124].

dipeptidyl-peptidase-4 (DPP4) is a membrane-bound dimeric peptidase that is widely expressed in different cell types and functions to degrade bioactive peptides. DPP4 can promote a variety of tumors and its abnormal expression is highly correlated with tumor invasion. Plasma membrane-associated DPP4-dependent lipid peroxidation is increased in human hepatocellular carcinoma cells, which brings about cellular ferroptosis. Mechanistically, it was found that P53 can inhibit ferroptosis in hepatocellular carcinoma cells by promoting the entry of DPP4 into the nucleus and forming a DPP4-P53 complex. Dissolution of this complex restores the sensitivity of hepatocellular carcinoma cells to erastin. In the absence of P53, DPP4 can also interact with NOX1 to form a complex that leads to increased lipid peroxidation and ferroptosis, and inhibition of DPP4 activity can significantly suppresses ferroptosis [125–127].

A recent study revealed that pretreatment of cells with Nutlin-3 delayed ferroptosis in various tumor cells (including hepatocellular carcinoma cells) and that the inhibition of ferroptosis was mainly dependent on a key target of P53-regulated transcription-CDKN1A (which can encode P21), but the exact mechanism is unclear and may be related to the accumulation of intracellular GSH leading to reduced cellular ferroptosis sensitivity related [128].

In conclusion, the role of P53 in ferroptosis is colorful and more in-depth studies are needed to explore potential therapeutic implications [128, 129].

### P62-Keap1-Nrf2-ARE

Nuclear factor erythroid 2-related factor 2 (Nrf2), is a key transcription factor in the antioxidant response. Under non-oxidative stress conditions, Nrf2 protein expression is low because Nrf2 binds to Keap1, mediating ubiquitination modifications that result in rapid proteasomal degradation of Nrf2 [130]. In contrast, under oxidative stress conditions, Cys273 and Cys288 cysteines on Keap1 protein are rapidly oxidized, leading to the loss of Keap1 protein-mediated ubiquitination modifications and the translocation of Nrf2 to the nucleus, where it binds to multiple AREs on DNA and initiates the transcriptional expression of multiple antioxidant proteins [131]. Under oxidative stress, P62 protein can regulate the protein stability of Nrf2 and the transcriptional activity of downstream AREs by directly interacting with KEAP1 [132]. Numerous studies have shown that the P62-Keap1-Nrf2 signaling pathway exerts an inhibitory role in ferroptosis in HCC cells [133].

Upon exposure of HCC cells to specific ferroptosis inducers (e.g., Sorafenib, erastin, etc.), the expression of P62 increases and prevents the degradation of Nrf2 protein by competitively binding to Keap1 protein, and promotes the entry of Nrf2 into the nucleus [134], where nuclear Nrf2 can bind to transcriptional co-activators such as Maf protein and activate a series of downstream ferroptosis-related proteins, especially antioxidant proteins transcription, including NADH Dehydrogenase, Quinone 1 (NQO1), heme oxygenase-1 (HO-1), and FTH1 [135–138]. NQO1 reduces ubiquinone to ubiquinol and directly inhibits the accumulation of ROS [139]. activation of HO-1 catalyzes the degradation of heme to ferrous iron and increases the amount of free iron, thereby increasing the susceptibility of cells to ferroptosis [140, 141]. FTH1 is a subunit of the major intracellular iron storage protein. When FTH1 is reduced, it causes intracellular iron overload, and iron overload promotes the production of large amounts of ROS by Fenton, consequently promoting cellular ferroptosis [142]. Expression of each of these genes prevents the aggregation of iron-dependent lipid peroxides. Inhibition of Nrf2 expression using shRNA resulted in reduced expression of NQO1, HO1, and FTH1 expression, promoting ferroptosis in erastin- and sorafenib-treated HCC cells [143, 144].

A study found that GSTZ1 can also affect the sensitivity of HCC cells to sorafenib-induced ferroptosis via the Nrf2 pathway. This study found that HCC cells with low GSTZ1 expression were highly tolerant to sorafenib [145]. While overexpression treatment of GSTZ1 in HCC cells could increase the characteristic products of ferroptosis induced by sorafenib, such as MDA and 4-HNE; while treatment with t-BHQ, an activator of Nrf2, could significantly reduce the expression of these products, inhibiting the extent of ferroptosis in HCC cells.

However, the role of Nrf2 in ferroptosis is complex and variable; on the one hand, inhibition of Nrf2 can cause ferroptosis in tumor cells, and on the other hand, activation of Nrf2 can also set off ferroptosis in tumor cells. Tumor cells have increased iron ion content compared to normal cells, and activation of Nrf2 can HO-1 activation, while excessive activation of HO-1 can cause cellular iron overload, induce cellular ferroptosis and exert cytotoxicity.

All these findings suggest that the stability and transcriptional activity of Nrf2 protein are critical in determining the effect of ferroptosis-targeted therapy in HCC cells.

### Rb

Rb proteins are essential members of the Rb protein family, which is primarily responsible for the cell cycle and hepatocarcinogenesis [146]. loss of Rb function is a common molecular event in primary HCC. At the animal level, Rb deficiency can also directly contribute to hepatocarcinogenesis [147, 148].

Louandre’s team delved into the interrelationship between Rb proteins and sorafenib-induced ferroptosis in HCC cells in in vivo and in vitro experiments [149]. Their results showed that reducing Rb protein expression using shRNA in the presence of sorafenib treatment increased the amount of ROS in the mitochondria of HCC cells to some extent, which increased the sensitivity of HCC cells to sorafenib-induced ferroptosis, probably because silencing Rb protein increased the expression of GPX4 mRNA in the cells, suggesting that Rb protein silencing resulted in HCC cellular susceptibility to sorafenib may be caused by an imbalance in cellular antioxidant capacity.

Therefore, Rb protein plays a considerable role in the progression of ferroptosis in HCC cells. However, it is not clear how useful the detection of Rb protein levels in HCC tissues is in predicting sorafenib treatment efficacy remains unclear.

### MT-1G

Metallothionein, MT is a family of intracellular proteins widely expressed by eukaryotic cells and divided into four subgroups (MT1–MT4) characterized by a common structure and a high cysteine content composition.

It has been discerned that MT1G can be used as a biomarker for altered redox metabolism in HCC cells. MT1G was most significantly upregulated in the presence of sorafenib, and the overall survival rate of HCC patients treated with sorafenib was negatively correlated with MT1 protein expression level in serum. MT-1G can promote the resistance of HCC to sorafenib and inhibit the expression of MT-1G in vivo or in vitro to enhance the anticancer activity of sorafenib. This effect of MT-1G is achieved by blocking the GSH depletion-mediated lipid peroxidation to inhibit ferroptosis in HCC cells, independent of iron ion content.

In a parallel study similarly indicated that sorafenib upregulated MT1G expression in HCC cells. Further studies revealed that MT1G is an additional target gene of Nrf2, and inhibition or knockdown of Nrf2 significantly inhibited MT1G expression and increased ROS production in HCC cells. Consequently, this heightened cellular sensitivity of cells to sorafenib-induced ferroptosis [150].

Thus, MT1G could be both a promising biomarker for predicting sorafenib efficacy and a potential therapeutic target for sensitizing sorafenib efficacy. MT-1G holds promise as a target to overcome the acquired resistance of HCC cells to sorafenib.

### S1R

The Sigma 1 receptor (S1R) is a membrane protein found in the central nervous system, liver, lung, and other organs and tumors.

It is commonly understood that S1R modulates ROS through Nrf2, serving as a cell protector in cells. When S1R was knocked out, Nrf2 expression was downregulated and KEAP1 expression was increased. Nrf2 can in turn regulate S1R gene expression, and in sorafenib-treated HCC cells, if Nrf2 is inactivated and intracellular ROS accumulation, S1R expression is upregulated to protect HCC cells from ferroptosis [151]. In addition, ferroptosis inducers such as erastin and Sorafenib significantly upregulated the protein expression level of S1R but not the mRNA level, suggesting a transcription-independent role of S1R in ferroptosis [152].

These findings imply that S1R may regulate ferroptosis by negative feedback through Nrf2-dependent and GPX4-dependent pathways at the same time, but the trigger conditions and the bridge connecting the two pathways remain to be discovered.

### CER

CER is a glycoprotein synthesized mainly by the liver and plays a key role in iron metabolism [153]. It has the effect of antioxidant, and has oxidase activity, and can catalyze the oxidation of polyphenol and polyamine substrates.

CER is able to regulate iron metabolism levels in HCC cells to modulate iron toxicity, and when protein levels are reduced, It leads to increased intracellular iron levels and accumulation of lipid peroxides, Then promotes erastin and RSL3-induced iron toxicity, which may be achieved by relying on FPN, The inhibitory effect of CER on ferroptosis was abolished when FPN expression was inhibited by shRNA [154]. This underlines the intricate involvement of CER in the regulation of iron-related cellular processes and its role in mitigating ferroptosis responses in HCC cells.

### ACSL4

Ferroptosis proceeds through a series of enzymatic reactions of lipid peroxidation. In a screen for key lipid peroxidases in iron toxicity, Acyl-CoA synthetase long-chain family member 4 (ACSL4) was shown to be perhaps indispensable as a key factor in promoting iron toxicity. It was found that ACSL4 expression in HCC cell lines was positively correlated with sorafenib sensitivity. The use of ACSL4 inhibitors or knockdown of ACSL4 in HCC cells significantly enhanced the anti-HCC efficacy of sorafenib. More importantly, the expression of ACSL4 in HCC cells does not change with sorafenib treatment and has a certain stability, which means that ACLS4 is a good biomarker for predicting the treatment of HCC by sorafenib through ferroptosis pathway [155]. ACSL4 is not only serves as a key executor but also functions as a reliable indicator of iron deposition [27].

### CISD1

CDGSH iron-sulfur domain 1 (CISD1) is an iron-containing mitochondrial outer membrane protein that is widely found in mitochondria-rich tissues such as liver and heart [156]. The main role of CISD1 is to regulate iron uptake in mitochondria and mitochondrial respiratory function. CISD1 deficiency leads to iron ion accumulation and subsequent mitochondrial oxidative damage [157].

Yuan et al. found that subferric ion content in HCC cells is augmented by erastin induction, and the expression of CISD1 is also increased simultaneously. Knockdown of CISD1 using shRNA increased the content of subferric ions in the mitochondria of HCC cells, subsequently increasing the extent of lipid peroxidation in the mitochondria and ultimately increasing the sensitivity of HCC cells to erastin-induced ferroptosis [158].

### HNF4A/HIC1

Human hepatocyte nuclear factor alpha (HNF4A) and Hypermethylated in cancer 1 (HIC1) are two transcription factors with opposite functions in the regulation of HCC growth [159]. HNF4A is upregulated in HCC and stimulates EGFR-mediated tumor proliferation response, thereby fostering HCC progression [160]. In contrast, HIC1 inhibits tumor growth, migration, and invasion [161, 162].

**Fig. 3:**
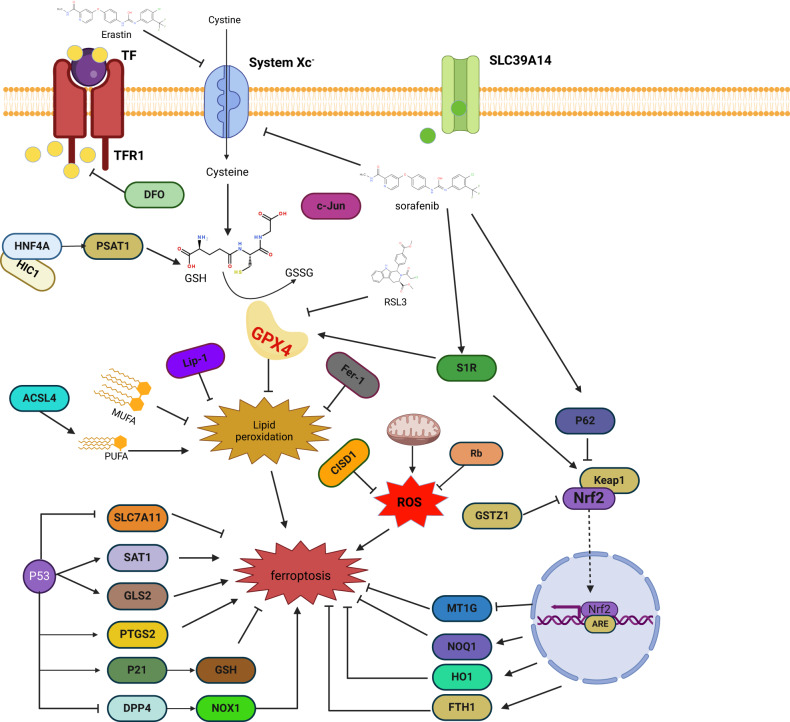
Ferroptosis regulatory network in HCC. Cystine enters the cell through the Xc^−^ system to be converted to GSH, and erastin and Sorafenib inhibit this pathway. While RSL3 can directly target GPX4. P53 regulates ferroptosis by affecting the transcriptional level of downstream SLC7A11, SAT1, GLS2, PTGS2, P21, DPP4, etc. Rb, CISD1 can directly regulate the intracellular ROS level. GSTZ1 and P62 are by affecting the entry of Nrf2 into the nucleus to affect the MT1G, NQO1, HO1, and FTH1 levels to regulate ferroptosis.

In studies targeting ferroptosis in HCC, HNF4A was found to transcriptionally regulate such as STMN1, CAPG, and RRM2 under the action of erastin, and to increase GSH production and thus inhibit ferroptosis by promoting PSAT1. In contrast, HIC1 can regulate such as HBA1, NNMT, PLIN4 and promote ferroptosis in HCC by repressing PSAT1 [163]. Therefore, breaking the balance between HNF4A and HIC1 may contribute to the treatment of HCC. (Table 2), (Fig. 3).

**Table 2:** Relevant genes regulating ferroptosis in HCC.

Target gene	Function	Regulatory mechanism	Validated experimental system
*P53*	Transcription factor	As transcription factors can promote ferroptosis by up-regulating the expression levels of the ferroptosis’s promoters SAT1, GLS, PTGS2 and down-regulating the ferroptosis’s repressor SLC7A11 [110, 118].	Clinical patients, animals, cells
*P62*	transcription factor	Regulation of Nrf2 entry into the nucleus by binding to Keap1 thereby initiating transcription of downstream antioxidant components [128, 158].	Clinical patients, animals, cells
*Rb*	suppressor gene	Increased ROS content in HCC cell mitochondria [121]	Clinical patients, animals, cells
*MT-1G*	transcription factor	Promoting ferroptosis by increasing intracellular ROS levels through oxidative stress [144].	Clinical patients, animals, cells
*S1R*	transcription factor	In part, it plays a regulatory role by affecting the Keap1/Nrf2 pathway, and on the other hand, it promotes ferroptosis by up-regulating iron metabolism-related genes FTH1 and TFRC1 [145].	animals, cells
*CER*	glycoprotein	Regulation of ferroptosis by modulating iron metabolism levels in hepatocellular carcinoma cells [159].	animals, cells
*ACSL4*	enzymes	Promoting ferroptosis by synthesizing polyunsaturated fatty acids as substrates for ferroptosis [119, 160].	animals, cells
*CISD1*	membrane protein	Increased oxidative damage in mitochondria by modulation of ferrous iron substituent content in mitochondria [153].	animals, cells
*HNF4A/HIC1*	transcription factor	Positive and negative feedback modulation of ferroptosis sensitivity [155, 156].	animals, cells

Summary of core targets in the ferroptosis regulatory network of hepatocellular carcinoma and their mechanisms of liver cancer treatment.

## Classical ferroptosis inducers in hepatocellular carcinoma

### Erastin

**Fig. 4:**
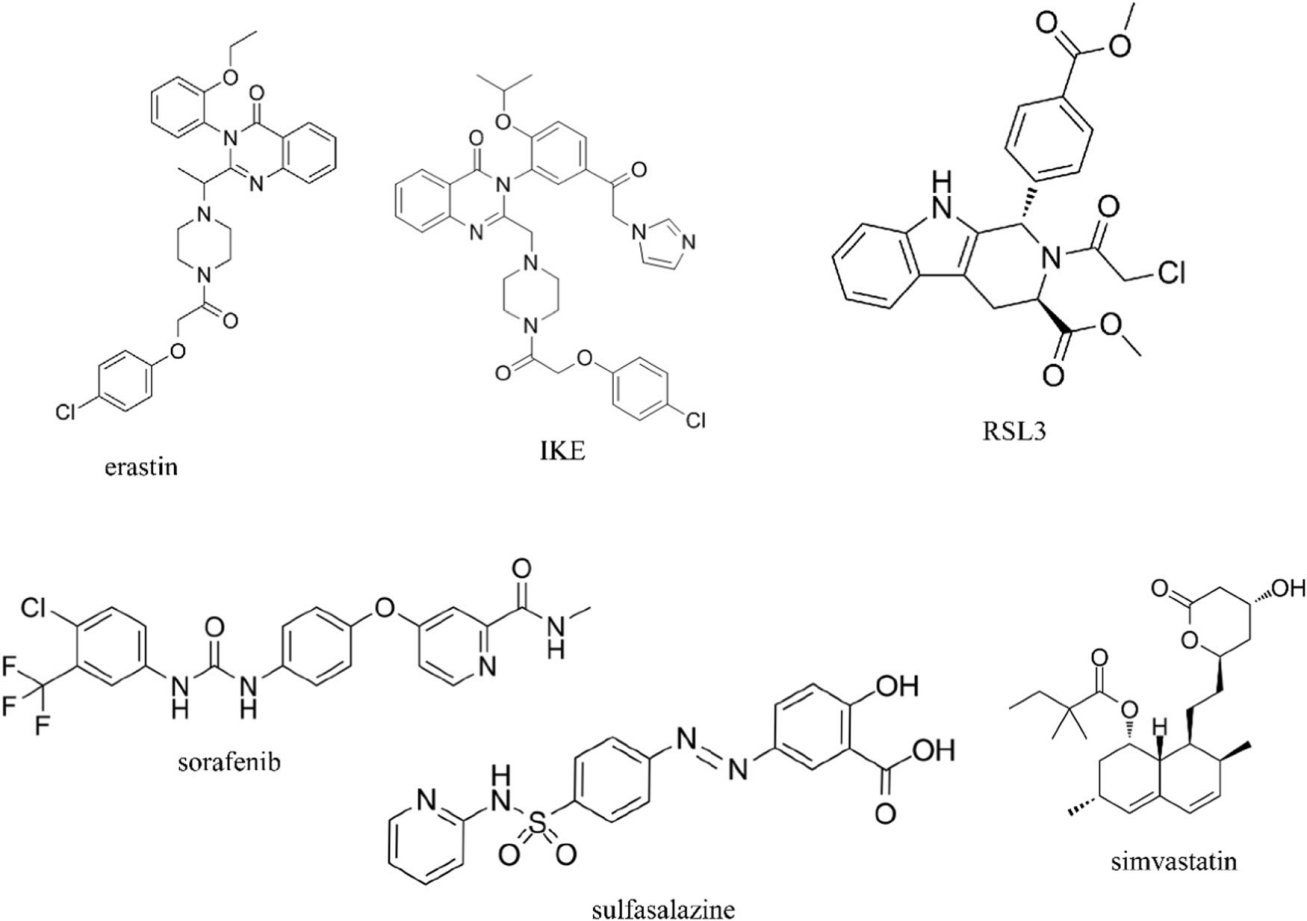
Chemical structural formulae of traditional ferroptosis inducers. Chemical structural formula of erastin, IKE, RSL3, sorafenib, sulfasalazine, and simvastatin.

A high-throughput screening of synthetic compounds for tumor cell killing identified a compound with RAS-selective activity: erastin. It was found to induce a non-apoptotic cell death. Subsequently, further studies revealed that erastin reduced GSH levels by directly inhibiting Systems Xc^−^ (Fig. 4). The quinazolinone backbone may be the main active moiety for its lethality, and other moieties could improve the inhibitory effect of erastin on Systems Xc^−^. The mitochondrial voltage-dependent anion system is also a molecular target of erastin. In addition to its ability to directly activate ferroptosis for hepatocellular carcinoma treatment, erastin also enhances the chemotherapeutic effects of certain conventional antitumor agents in hepatocellular carcinoma cell lines. erastin could enhance the clinical efficacy of PD1/L1 by affecting the polarization of tumor-forming associated macrophages [164]. Aspirin induced a significant ferroptosis response in HepG2 and Huh7 cells, which was enhanced by the ferroptosis inducer erastin [134].

As a classical ferroptosis inducer, erastin has been used by researchers as a standard reference for testing novel compounds or ferroptosis inducers of existing drugs.

### IKE

Although erastin has a good inhibitory effect on Systems Xc^−^, its low water solubility and metabolic instability seriously affect its in vivo application. Imidazolidinone (IKE), a derivative based on the improved structure of erastin, further improved the aqueous solubility and anticancer properties of the prototype, with three times the solubility of erastin and a 50-fold reduction in the LC50 for tumor cells [165, 166].

### RSL3

In a gene-selective screening assay of more than 40,000 compounds related to cell proliferation, two small molecule compounds, RSL3 and RSL5, were screened for high lethality in the presence of oncogenic RAS [167]. Further experiments revealed that RSL3 could induce ferroptosis by directly targeting the inhibition of GPX4 [168], however, it has also been suggested that RSL3 is not an inhibitor of GPX4 but TXNRD1 [169]. the chloroacetamide portion of the RSL3 structure was essential for its bioactivity. RSL3 induced ferroptosis in a wide range of hepatocellular carcinoma cell lines, including all of them, and RSL3 could also enhance the sensitivity of tumors to ferroptosis in the presence of IFN-γ [170].

### Sorafenib

Sorafenib is a multikinase inhibitor that has been widely used in patients with clinically advanced hepatocellular carcinoma. Sorafenib can prolong the survival of patients with hepatocellular carcinoma. Previous studies have suggested that the action of sorafenib in the treatment of hepatocellular carcinoma is mainly due to its multikinase inhibitory effect, which leads to the inhibition of cell proliferation, angiogenesis and the promotion of apoptosis of tumor cells. It was found that the toxic effect of sorafenib on hepatocellular carcinoma cells was partly dependent on ferroptosis rather than apoptosis, and this toxic effect was also not dependent on its multikinase inhibitory activity, but induced ferroptosis by inducing iron aggregation and lipid peroxidation stress in hepatocellular carcinoma cells, and that depletion of stored iron in hepatocellular carcinoma cells by use of iron chelating agents could effectively weaken the cytotoxic effect of sorafenib. Further studies revealed that sorafenib induces ferroptosis by inhibiting SLC7A11 transporter activity on the cell membrane, thereby reducing cystine content in hepatocellular carcinoma cells, which in turn leads to insufficient GSH synthesis and diminished GPX4 activity. Hepatocellular carcinoma patients treated with clinical application of sorafenib tend to develop drug resistance quickly, thus affecting the prognosis. As mentioned above, Nrf2, Rb, MT-1G and SIR are involved in the regulation of the sensitivity of ferroptosis induced by sorafenib through the corresponding pathways, thus inducing the emergence of drug resistance. From a new perspective, targeting the relevant inhibitor pathways to induce ferroptosis can effectively improve the drug resistance of sorafenib.

### Sulfasalazine

SAS is an anti-inflammatory drug which has been approved by the U.S. Food and Drug Administration for a long time, is a first-line treatment for rheumatoid arthritis. SAS induces ferroptosis by inhibiting the Xc-system in a manner similar to erastin. However, compared to erastin, SAS is much less effective in inducing ferroptosis [171]. SAS has been shown to induce ferroptosis in a variety of tumor cells and may also be used in combination with other liver cancer therapies to improve treatment efficacy.

### Iron-containing statins

Classic iron-containing statins include pravastatin sodium, lovastatin, and simvastatin. Statins, chemical inhibitors of HMG-CoA reductase, are a class of lipid-lowering drugs that are effective in reducing the prevalence of cardiovascular disease and mortality [172]. HMG-CoA reductase promotes mevalonate synthesis, and statins can inhibit MVA synthesis to block CoQ10 synthesis, which is a substrate for lipid peroxidation by the ferroptosis inhibitors FSP1 and DHODH, and statins can also directly affect GPX4 activity to promote ferroptosis. In addition, it has been found that atorvastatin-treated tissues have elevated levels of ROS, MDA, and Fe^2+^ along with decreased expression of GSH and GPX4, suggesting the presence of a ferroptosis response. The suppression of the HMG-CoA reductase by statins leads to decreased expression of glutathione peroxidase 4 (GPX4), a key regulator of lipid peroxidation, which in turn results in lipid ROS production and induction of ferroptosis. Experimental data suggest that statins may act as anti-cancer drugs by enhancing tumor cells’ ferroptosis [173, 174].

### Summary and outlook

Ferroptosis, as an emerging mode of cell death, participates in the development of HCC and may play a key role in both the diagnosis prevention treatment and prognosis of HCC. Although ferroptosis has opened a new horizon in the basic field in recent years, translation into specific clinical treatments is yet to be achieved.

It is believed that as ferroptosis-related research progresses and these key questions are answered, targeting ferroptosis to treat HCC, especially in combination with immunotherapy, may overcome immunotherapy resistance and benefit more HCC patients.
